# Prevalence of Developmental Delay in Apparently Normal Preschool Children in Isfahan,Central Iran

**Published:** 2015

**Authors:** Omid YAGHINI, Roya KELISHADI, Mojtaba KEIKHA, Negar NIKNAM, Saeid SADEGHI, Efat NAJAFPOUR, Mohammadreza GHAZAVI

**Affiliations:** 1 Faculty of Medicine, Isfahan University of Medical Sciences, Isfahan, Iran; 2 Child Growth and Development Research Center, Research Institute for Primordial Prevention of Noncommunicable Disease, Isfahan University of Medical Sciences, Isfahan, Iran; 3 Welfare Organization, Isfahan, Iran

**Keywords:** Developmental delay, Developmental screening, Child development, Iran

## Abstract

**Objective:**

Developmental delay screening is essential in pediatric medicine. The purpose of this study was to estimate the developmental delay in apparently normal children at entry to kindergarten.

**Materials & Methods:**

In this cross- sectional study conducted in 2013, the developmental status of a sample of children who entered to kindergarten at the age of 4-60 months were evaluated by the Persian version of ages and stages questionnaires (ASQ) in Isfahan county, central Iran.

**Results:**

Totally 680 children were enrolled, 11.8% of them were suspected to delayed in at least one domain and 1.3% and 1.2% in two and three domains, respectively. Developmental delay was in the following items: 5% in problem solving; 4.9% in fine motor; 3.2% in gross motor, 2.2% and 1.2% in personal – social and communication domains, respectively.

**Conclusion:**

Considerable proportions of apparently normal children who are entering kindergarten had developmental delay, which could be detected by evaluation with appropriate screening tools.

## Introduction

Poverty, poor health and hygiene, malnutrition, and deficient care are including risk factors associated with functional impairment and cognitive developmental delay in infants and children in low-and middle-income countries (1, 2). According to global statistics, about 5 to 16% of children have developmental disorders (3, 4). Approximately 30- 50% of these disorders are not identified until school age and therefore could not be treated (5). The importance of early detection of disorder in safety, health and welfare of the child and his family has been proved. The American Pediatric Academic Society recommendations are using validated methods and tools for early diagnosis and treatment of developmental disorders in children. When there are only limited clinical diagnosis and clinical judgment available, there could be only about 30% of children with developmental problems and disorders diagnosed before school age (6). Screening for developmental disorders in children with undiagnosed developmental disability and delay is likely to be helpful in diagnosis of the issue for pediatricians (7) . Parent-based developmental screening tools are a brief assessment to identify children who supposed to get diagnostic evaluation that is more precise (3, 8). Using maternal reports on child development has shown that the parent’s information about their child’s ability is much valued for prediction of developmental disorders (9, 10). A screening method for diagnosing of developmental disorders on time is to use parents’ collaboration and screening questionnaires, which should be filled by parents or physicians (9). Parents have accurate information about their child’s development (11, 12) and their comments have high credibility and lead to increasing in the diagnosis of disorder (13-15). One of the ways that is a questionnaire filled by parents called the (ASQ2), Ages and Stages Questionnaires are widely used nowadays (16-18). The sensitivity and specificity of ASQ measured in different studies, respectively are, 75% and 95% (19). The questionnaire was translated into Persian and its validity and reliability was approved (20). The questionnaire is used for ages 4-60 months in five different domains of communication, fine motor, gross motor, problem solving, and personal-social skills (21). As secondary prevention is the result of optimal screening and given the importance of optimal development of children and its impact on individual and social life, infants and children need developmental screening methods. Such screening should be done by using a simple, low- cost, and applicable tools to identify potential problems faster and better and then to take timely interventional treatments for these individuals . The purpose of this study was to verify the importance of screening of all infants and children for developmental disorders (delay) at the time of nursery admission before they enter to the school.

## Materials & Methods

This cross-sectional study was conducted in 2013 in Isfahan, central Iran. We screened the developmental delay in infants and children aged less than 60 months entering kindergarten by using ASQ2 questionnaires (Iranian version). Along the cooperation and coordination of Isfahan welfare society with acquisition of necessary permits, the number of the city kindergartens and their locations were collected. The city was divided into five areas of center, west, east, north and south. By considering the frequency of developmental disorders as 5-16% (3, 4) and by using the following equation: n=z2 p (1-p)/d2 ; z=1.95 ;p=0.2 ; d=0.15 p}, the sample size was calculated as 682. To compensate possible loss in cases, we increased this number to 1000. In late September and early October, the questionnaires were filled in done by parents who were informed about ASQ2. A written informed consent, which was approved by ethics committee of Isfahan University of medical sciences, was also filled in by the parents to allow investigators in using their children in formation for the study. After choosing kindergartens and their population, all children of that kindergarten were enrolled in the study. However, given the loss in samples volume and lack of response, approximately 680 samples were used in the final data. Inclusion criteria included: 1) Children aged 4 to 60 months 2) Parents cooperation, and exclusion criteria were: 1) Known developmental delay in children, 2) Mothers refrain from entering and cooperating in the study. The data collection procedure was based on completion of questionnaires by parents in the selected kindergartens after clarifying the purpose of research project to them and explaining how to complete the questionnaires. Questionnaire was along with an information sheet and a consent sheet that contained basic information of the project. After completing the questionnaires, entering data were analyzed by (SPSS) software version 20 (Chicago, IL, USA) and for data description, central tendency and dispersion of data and graphs, and tables were used. Error of 5% significance level in all tests was considered.

## Results

Among participants, the questionnaires of 15 cases were not fully completed, so were not included in the analysis. Overall, 680 children who had unknown developmental disorders were screened by ASQ2 questionnaires. 

**Table 1:** Frequency of Participant According by Age

**Age (month)**	**Frequency**	**Percent**
36	98	14.4
42	119	17.5
48	136	20.0
54	150	22.1
60	164	24.1
Missing	13	1.9
Total	680	100

Eighty children (11.8%) were unable to get appropriate scores in the area of development and were reported as failed ASQ2. Because the developmental status was assessed in five areas, the disorder frequency in each area was as follows: incidence of developmental delay in problem solving 34 (5%), fine motor 33 (4.9%), gross motor 22 (3.2%), Personal-social skills 15 (2.2%) and Communication 8 cases (1.2%). Sixty-one patients (9%) had defect in one domain and 9 patients (1.3%) faced disorders in two areas and 8 (1.2%) children had developmental delays in three domains. Table 2 shows frequency of dysfunction according to type (scope) of defects.

**Table 2:** Frequency of Dysfunction According to Type (Scope) of Defects*

**Scope**	**Number of subject with dysfunction**
**Communication**	8
**gross motor**	22
**fine motor**	33
**problem solving**	34
**Personal-social skills**	15

* Since each person can have more than one scope of defects, the sum of frequency is greater than 80.

Table 3 shows frequency of defects according by age of participant. Chi-square test showed that between different age ranges and number of defects was significant differences P<0.000.

**Table 3:** Frequency of Defects According by Age of Participant

		Age (month)					Total	*P* value
		36	42	48	54	60		
screen	+	15	6	27	0	32	80	0.000
	-	83	113	109	150	132	587	
Total		98	119	136	150	164	667	

* Chi-square test showed that between different age ranges and number of defects there was significant differences (*P*<0.000)

Forty six percent of the population was male. There were no significant differences in the prevalence of developmental disorder in boys and girls (P= 0.057). Table 4 shows demographic characteristic of participant.

**Table 4:** Demographic Characteristic of Participant

	**Boy**	**Girl**
**Birth weight(gram)** 1	3175(500)	3053(474)
**Number of household children**	**Frequency**	**Percent**
1	324	47.6
2	241	35.4
3	49	7.2
4	5	0.7
5	1	0.1
**Mothers education**	**Frequency**	**Percent**
Illiterate	4	0.6
Primary	36	5.3
Intermediate	70	10.3
Diploma	223	32.8
Under graduate	230	33.8
Post graduate	43	6.3
**Fathers education**	**Frequency**	**Percent**
Illiterate	3	0.4
Primary	55	8.1
Intermediate	106	15.6
Diploma	220	32.4
Under graduate	177	26.0
Post graduate	55	8.1

1 Data are presented as mean (SD)

The type of feeding (formula or breast milk), history of birth asphyxia, febrile seizures, brain infections and birth complications showed no obvious difference between the two groups with normal and abnormal ASQ. t-test showed no statistically significant difference in birth weight between the two groups of children. Mann-Whitney test showed significant association between a developmental disorder and the father educational status (P= 0.014). The less educated father is more likely to have a child with developmental delay but there was no significant difference between developmental disorders and maternal education or family income.

## Discussion

Our study showed that about 11.8% of these children had developmental disorders while they were considered normal and got normal education, although they demand to be educated exceptionally. Several studies of developmental screening in Iran and other parts of the world have used this questionnaire for screening. Sajedi et al. in Tehran, evaluated the prevalence of developmental disorders by using the ASQ Questionnaire (22). In this study, the prevalence of developmental disorder (using ASQ) was 3.69% to 4.31%, and among developmental skills areas the high frequency was related to fine motor and personal-social skills. However, this study was conducted in several cities, site of implementation was in health centers, the age was lower than our study, and no other criteria were determined for entry and exit but the age. However, in our study the prevalence of disorders in five domains has been 1.2% to 5%; that among developmental domains, most evolutionary fields were related to the areas of problem solving and fine motor. Darreh et al. in Arak have examined the condition of children 4 to 60 months with history of neonatal intensive care unit admission (23). They screened using ASQ2 in 5 domains of communication, fine motor, gross motor, problem solving and personal-social skills, respectively. 20.2%, 19.3%, 17.5%, 8.8% and 16.7% of children were abnormal. There was no relation between sex, birth weight and length of hospital stay in the previous study (23). The high percentage of patients , in this study may be due to sampling of high-risk group because low-birth weight infants especially those with weight less than 1500 grams and/or with any experience of neonatal intensive care unit admission are exposed to having developmental disorders. The difference in sex has no effect on the result of this study like the others. Yaghini and colleagues in 2012 studied 800 six-month old infants referring for vaccination using ASQ tests (24). Of these, 10.5% failed in screening at least in one domain. These figures were in consistent with what we found in the current study. Regardless of the different ages of children at the time of testing, which can be a determining factor no other criteria were considered and children were randomly selected (24). However, in this study, when these children (those studied at 6 months), at 24 months were studied for the second time, 4% of them had developmental disorders that might be due to different levels of sensitivity and specificity of the test at different ages. But the same result of 10%, is obtained in other studies (25, 26) . In this study, no association was found between ASQ domains and birth weight, premature birth, perinatal developmental disorders. Meanwhile Karimi and colleagues in a cross-sectional study assessed the developmental status of children with low birth weight (LBW) (27). According to exclusion criteria of the study, children who had significant perinatal events such as birth asphyxia were removed from the study. In this study, areas of gross motor, fine motor and problem solving among children with LBW compared with normal children had a higher frequency. This difference was significant but in our study due to the low population of LBW children, this evaluation was not possible. Low-educated mother, premature birth (premature) and multiple deliveries were related factors to disorders that in our study (27). In case of fathers’ educational level, its association with developmental disorder was seen in our study. However, in some studies, rates of these disorders were higher than our study. In a study two screening tools were compared and 18% were failed in ASQ2 (28). In another study, 27% of children suffered from developmental delay based on ASQ Questionnaire (29). In Yang et al. study 25.4% of children were classified as failed or positive (30). The omission of typical developmental disorder cases at the beginning of our study could be the reason of this difference. In our study, 1,000 people were selected initially, but only 680 of them remained to the end of the study, that it could disturb the appropriate conclusion. In conclusion, a significant proportion of apparently normal children had positive screening result for developmental delay if they had been evaluated with appropriate screening tools. Since the follow-up and interventions before the school time could affect the educational status and future of these children, it is recommended to evaluate all the children of that age, 3 to 4 years, by appropriate tools. 

## Notes

**Acknowledgements** We thank to Isfahan Welfare Organization for their support and cooperation in this study.
